# Metabolic Profile of Oral Squamous Carcinoma Cell Lines Relies on a Higher Demand of Lipid Metabolism in Metastatic Cells

**DOI:** 10.3389/fonc.2018.00013

**Published:** 2018-02-02

**Authors:** Ana Carolina B. Sant’Anna-Silva, Gilson C. Santos, Samir P. Costa Campos, André Marco Oliveira Gomes, Juan Alberto Pérez-Valencia, Franklin David Rumjanek

**Affiliations:** ^1^Instituto de Bioquímica Médica Leopoldo de Meis, Centro de Ciências da Saúde, Universidade Federal do Rio de Janeiro, Rio de Janeiro, Brazil; ^2^Centro Nacional de Biologia Estrutural e Bioimagem I (CENABIO I)/Centro Nacional de Ressonância Magnética Nuclear (CNRMN), Laboratório de Ressonância Magnética Nuclear de Biomoléculas (bioNMR), Universidade Federal do Rio de Janeiro, Rio de Janeiro, Brazil

**Keywords:** oral squamous cell carcinoma, metastasis, cancer progression, nuclear magnetic resonance, metabolomics, metabolic reprogramming

## Abstract

Tumor cells are subjected to a broad range of selective pressures. As a result of the imposed stress, subpopulations of surviving cells exhibit individual biochemical phenotypes that reflect metabolic reprograming. The present work aimed at investigating metabolic parameters of cells displaying increasing degrees of metastatic potential. The metabolites present in cell extracts fraction of tongue fibroblasts and of cell lines derived from human tongue squamous cell carcinoma lineages displaying increasing metastatic potential (SCC9 ZsG, LN1 and LN2) were analyzed by ^1^H NMR (nuclear magnetic resonance) spectroscopy. Living, intact cells were also examined by the non-invasive method of fluorescence lifetime imaging microscopy (FLIM) based on the auto fluorescence of endogenous NADH. The cell lines reproducibly exhibited distinct metabolic profiles confirmed by Partial Least-Square Discriminant Analysis (PLS-DA) of the spectra. Measurement of endogenous free and bound NAD(P)H relative concentrations in the intact cell lines showed that ZsG and LN1 cells displayed high heterogeneity in the energy metabolism, indicating that the cells would oscillate between glycolysis and oxidative metabolism depending on the microenvironment’s composition. However, LN2 cells appeared to have more contributions to the oxidative status, displaying a lower NAD(P)H free/bound ratio. Functional experiments of energy metabolism, mitochondrial physiology, and proliferation assays revealed that all lineages exhibited similar energy features, although resorting to different bioenergetics strategies to face metabolic demands. These differentiated functions may also promote metastasis. We propose that lipid metabolism is related to the increased invasiveness as a result of the accumulation of malonate, methyl malonic acid, n-acetyl and unsaturated fatty acids (CH_2_)_n_ in parallel with the metastatic potential progression, thus suggesting that the NAD(P)H reflected the lipid catabolic/anabolic pathways.

## Introduction

Oral squamous cell carcinoma (OSCC) is the most prevalent type of head and neck squamous cell carcinoma frequently leading to metastasis to the neck lymph nodes (1). OSCC evolves from high proliferation rates of the oral squamous epithelium ultimately forming an *in situ* carcinoma (2, 3) followed by metastasis and a high lethality rate (4, 5).

Compared to normal cells, cancer cells have been shown to display a reprogrammed metabolism resulting from the specific energy demands imposed by growth factor signaling (6, 7). Furthermore, in the case of metastatic cells, migration and colonization of distant tissues also contribute to the extra energy burden. Thus, we envision metastatic cells as a subpopulation of cells that were selected in terms of a fine-tuned coordination that integrates nutrient uptake, anabolic, and catabolic processes. In addition, the microenvironment is variable insofar as the tumor anatomy is concerned. Whereas glucose, glutamine, and oxygen are freely available for those cells located on the surface of the tumor mass, the inner layers of cells are confronted by a radically different milieu characterized by paucity of nutrients and by hypoxia (8, 9). Consequently, these constraints introduce selective pressures that will reward metabolic plasticity. Those cells that can adjust to the different environments in the tumors will either thrive locally or eventually become detached and give rise to potentially metastatic cells. Successful adjustment can be achieved by gain of function through the concerted activation of expression of key enzymes that affect the metabolic flux and proliferative pathways as well as genes involved in the acquisition of resistance to anoikis through suppression of apoptotic programs. However, it is important to bear in mind that the metastatic phenotype probably results from non-adaptive innovation, that is, through the integration of pre-existing signaling pathways. By becoming manifest, these pathways confer different properties that enable cells to survive in an otherwise incompatible microenvironment (10–12).

Recently, the metabolomic approach using nuclear magnetic resonance (NMR) has become increasingly more informative. The availability of metabolomic data has been very useful for unraveling the metabolic pathways of several types of cancer as well as the biochemical features pertaining to metastasis (13–15). The main advantage of metabolomics rests on its ability to instantly and globally analyze metabolites quantitatively and qualitatively so that not only the involved pathways can be highlighted, but also their fluxes could be deduced (16, 17). Likewise, two-photon fluorescence lifetime imaging microscopy (FLIM), a non-invasive technique, has been successfully used to probe intact living cells in order to investigate their metabolism, thus affording a snapshot of their energy status. Experimentally, the auto fluorescence generated by both NADH and NADPH has been used to investigate the mitochondrial redox state and hence the energy producing pathways (18–20).

In the present study, we performed ^1^H NMR and FLIM determinations combined with functional experiments in order to evaluate the metabolic alterations that may be relevant to the metastatic phenotypes of tongue squamous cells carcinoma (SCC) cells.

## Material and Methods

### Cell Lines

In the present study, cell lines developed and isolated from squamous cellular carcinoma SCC-9 (ATCC CRL-1629) by Agostini et al. (21) were used. The first cell line produced named SCC-9 ZsGreen stably expresses a green fluorescent zebrafish plasmid (ZsG). The paper describes how SCC-9 cells were inoculated into the footpads of BALB/c nude mice and were recovered as LN1 cells, the first metastatic generation. Another round of inoculation of LN1 cells produced LN2 cells, the second metastatic generation. Normal fibroblasts isolated from biopsies (3) were a gift by Dr. Ricardo Colleta from Department of Oral Diagnosis (School of Dentistry of Piracicaba, State University of Campinas, Brazil).

### Cell Culture

For SCC-9 derived cells, Dulbecco’s Modified Eagle Medium: nutrient Mixture F12 (DMEM/F12; Gibco^®^, Life Technologies™, USA) was used. Media were supplemented with 10% fetal bovine serum (FBS—Cultilab, Brasil) and hydrocortisone 400 ng/ml (Sigma-Aldrich, USA). For fibroblasts, we used Dulbecco’s Modified Eagle Medium (DMEM *low*; Gibco^®^, Life Technologies™, USA) supplemented with 10% *donor bovine serum* (DBS; Gibco^®^, Life Technologies™, USA) and 1% penicillin–streptomycin. 1.1 × 10^6^ cells of ZsG, LN1, LN2, and fibroblasts were transferred to 60.1 cm^2^ Petri dishes and maintained for 48 h in an incubator series 8000 water-jacketed CO_2_ (Thermo Scientific), with 5% of CO_2_ humidity atmosphere. At least four independent biological replicates of each cell line were used for experimental analysis. All cell lines were genotyped and tested free for *Mycoplasma* sp. infection using polymerase chain reaction.

### NMR Metabolomics

ZsG, LN1, and LN2 cells were cultivated in DMEM/F12 and fibroblasts in DMEM low until 80% confluence. Then, ~1.2 × 10^7^ cells from each replicate were trypsinized, centrifuged, and all the cell pellets were normalized to ~30 mg for metabolite extraction. We adapted the protocol for polar phase extraction according to adaptations of the method of Bligh and Dyer biphasic extraction previously described (22). Briefly, cells were extracted with methanol/chloroform/water (2:1:0.8), vortexed for 2 min after each addition, and homogenized during 30 min in a shaker in an ice bath. The polar aqueous phase (supernatant) was centrifuged at 4,600 *g* for 20 min at 15°C, the supernatant was dried overnight under vacuum (SpeedVac) and stored at −80°C until used. The samples were suspended in 50 mM phosphate buffer, pH 7.4, containing 10% of D_2_O and 0.1 mM of 4,4-dimethyl-4-silapentane-1-sulfonic acid (DSS) for chemical shift reference.

All spectra were acquired in a Bruker Avance III HD spectrometer running at 500.13 MHz for ^1^H at 298 K. One dimensional spectra were acquired with excitation sculpting for water saturation, 20 ppm spectral width, 1.74 s relaxation delay, 64 K points, and 3 K accumulations. For assignments, we used ^1^H-^1^H TOCSY and pJRES. Spectra were aligned and processed in TOPSPIN 3.5 (Bruker-Biospin) and exported to AMIX (Bruker-Biospin), normalized by DSS intensity as internal reference, 0.02 ppm binned, and finally water and DSS signal were excluded. The peak intensities were used for the relative metabolite quantification in each sample.

For statistical analysis, datasets of at least four independent samples of each ZsG, LN1, LN2, and fibroblasts cell culture were collected. For the univariate analysis, we used multiple *t*-test with two-stage setup method of Benjamini–Krieger–Yekutieli, with false discovery rate (FDR) of 5%, with approach, assuming consistent SD, on GraphPad Prism 6.0. For multivariate analysis, we used Metaboanalyst 3.0 (23) with default practice (skip missing value imputation), filtered data with median intensity value (due to large amount of data), and normalized by sum of intensities. In addition, we applied the pareto scaling to reduce the impact of the buckets with high prevalence in each comparison [signaled as threonine, threitol, and (CH_2_)n] in the statistical analysis and to keep the data structure intact. Multivariate methods were used to compare all clusters (four groups) or paired groups (fibroblasts vs. ZsG; ZsG vs. LN1; LN1 vs. LN2) by means of principal component analysis and partial least-square discriminant analysis (PLS-DA). To validate class discrimination and avoid overfitting, we used a permutation test (1,000 permutations) based on separation distance, B/W-ratio, and cross-validation by the leave-one-out method (24, 25). We also calculated the variable importance in the projection (VIP-score) to analyze the ranking of the most important metabolites in separation groups and the PLS-regression coefficients for components 1, 2, and 3.

REACTOME free software (26) was used to determine the related metabolic pathways to metabolites accumulated in each comparison. Briefly, we listed all the related enzymes that produce each metabolite by manual curation, and it was analyzed into REACTOME using *Homo sapiens* as background, showing the corresponding metabolic pathways. FDR correction was used with significance of 0.05.

### Fluorescence Lifetime Imaging

The autofluorescence lifetime images of isolate cultured cells were acquired using a laser scanning confocal fluorescence microscope Nikon Eclipse TE2000-U equipped with a Spectra-Physics MaiTai HP Laser (Spectra-Physics). The microscope is coupled to a fluorescence correlation spectrometer Alba Flim (ISS, Inc.) and data were collected by VistaVision software (ISS, Inc.) and analyzed by SimFCS 4 software (27). The samples were excited by two photons at 740 nm using a 60×/1.20 Plan-Apochromat water immersion objective lens (Nikon). The emission was detected on an Avalanche Photodiode detector, through a bypass filter 450 nm. A total of 100 frames from five different fields of each sample were collected. The image size acquired was 256 × 256 and the pixel dwell time was 40 µs. The calibration was done using a solution of 250 µM of NADH in water. The fluorescence lifetime was calculated by the phasor approach. Briefly, through Fast Fourier Transformation, the fluorescence decay in each pixel is plotted on the phasor plot by the coordinates G and S. The distribution of phasor points reveals areas of different lifetimes within the universal semicircle (28). Free and bound NADH have lifetimes of 0.38 and 3.4 ns, respectively, and their fluorescence intensity decays are plotted in the universal semicircle (29, 30).

### High-Resolution Respirometry

High-resolution respirometry (Oroboros Oxygraph-2k) was performed to evaluate the oxygen consumption of intact cells, as previously described (31). 10^6^ cells/ml of ZsG, LN1 and LN2 cells were cultivated separately and suspended in culture medium (DMEM/F12) without fetal bovine serum and phenol red. Then, cells were placed into a respiration chamber until the steady-state respiratory flux was attained (~10 min). Subsequently, the following parameters were measured: ATP synthase independent respiration in the presence of oligomycin (oligo) allowing the observation of oxygen consumption uncoupled to ATP synthesis (“leak”); content of oxygen consumption inhibited by the presence of oligomycin (“coupled”); titration with carbonyl cyanide-*4*-(trifluoromethoxy) phenylhydrazone (FCCP) to assess the maximal oxygen consumption rates once it stimulates the electron transfer system efficiency (“ETS”); residual oxygen consumption (“ROX”) in the presence of rotenone (NADH dehydrogenase inhibitor) and antimycin A (AA) (cytochrome *bc_1_* inhibitor). Data acquisition and analysis were carried out with DatLab 5.1 software (Oroboros Instruments, Innsbruck, Austria).

### Lactate Release in Culture Media

After 24 h of incubation with completed DMEM/F12 + 10% FBS and hydrocortisone 400 ng/ml, the culture medium was replaced by fresh DMEM/F12 without phenol red and FBS. Aliquots from the culture medium were collected at 0 and 60 min of incubation to evaluate lactate release through enzymatic assay. The lactate assay was performed in hydrazine/glycine buffer, pH 9.2, containing 5 mg/ml β-NAD^+^ and 15 U/ml lactate dehydrogenase (LDH; Sigma-Aldrich). NADH absorbance was monitored in a microplate reader (SpectraMax M5, Molecular Devices) at 340 nm.

### Proliferation Assay

Proliferation assays were performed using Sulforhodamine B (SRB) colorimetric assays, as previously described (32). Briefly, cells were placed into 96-well plates. After each endpoint (0, 24, 48, and 72 h), the culture medium was removed and cells were fixed with 10% trichloroacetic acid for 1 h at 25°C. The plates were then washed with distilled water and incubated with SRB solution (1% in acetic acid) for 15 min. After rinsing with 1% acetic acid, the cell monolayers were dried and the proteins were solubilized with Tris pH 10.4. Absorbance was measured at 490 nm using a spectrophotometer (SpectraMax Plus 384, Molecular Devices).

### Statistical Analysis

Besides metabolomics, all the experiments were plotted as means ± SD for *n* independent experiments. Statistical significance to evaluate two groups was determined by the unpaired *t*-test, one-way ANOVA, and Dunnett posttest. Univariate analysis was done by multiple *t*-test, two-way ANOVA, and posttest Holm–Sidak. All set at alpha = 0.05. The graphs were generated by GraphPad Prism version 6.0 for Windows (GraphPad Software, La Jolla, CA, USA) and Excel (Microsoft Corporation*^®^*) with 95% of confidence level.

## Results

### Metabolic Profiles of Normal, Tumor, and Metastatic Cells

**Figure 1:**
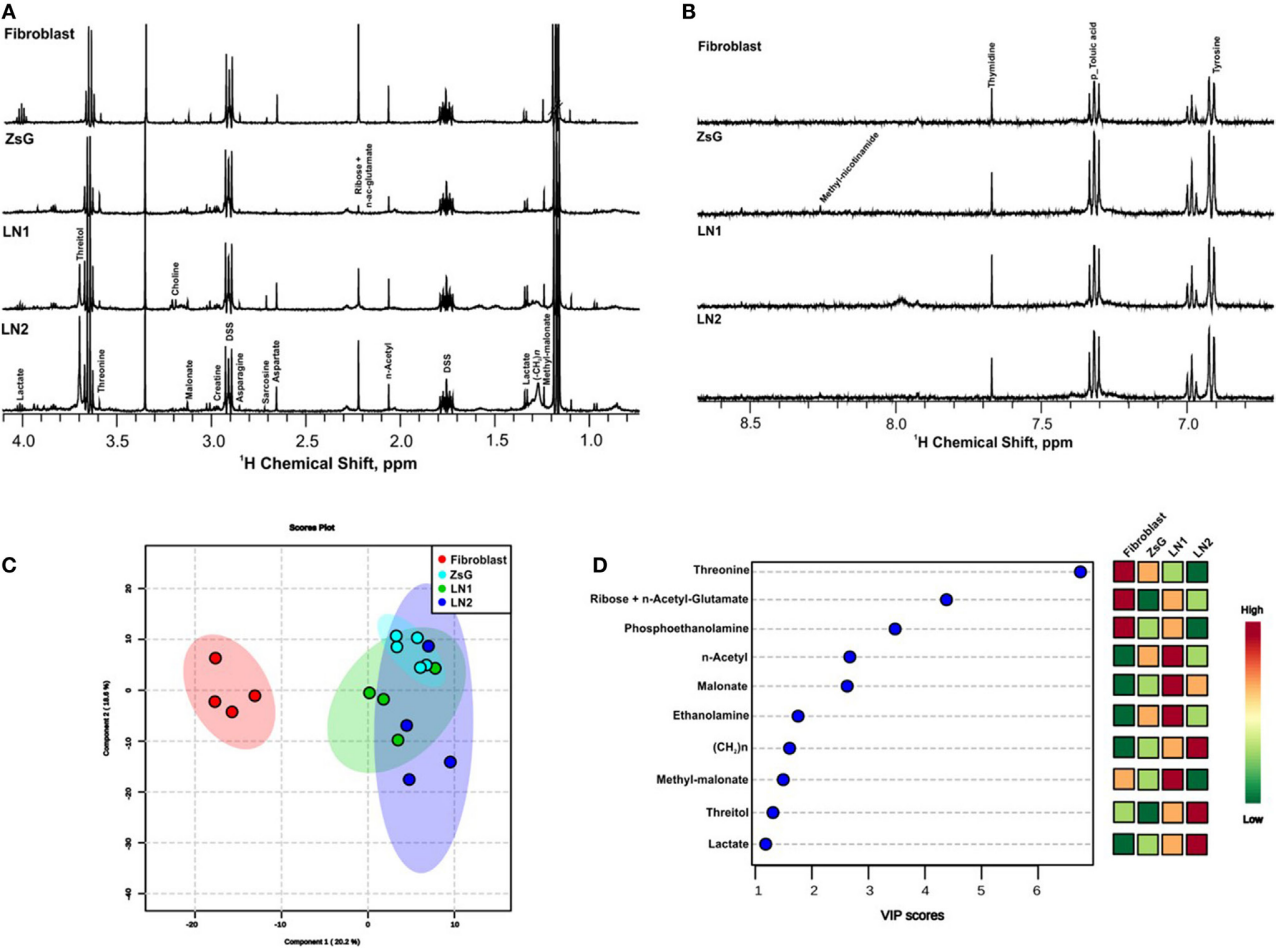
Metabolic profiles of normal, tumor, and metastatic cells. 1D ^1^H nuclear magnetic resonance representative spectra from fibroblast, ZsG, LN1, and LN2 cells polar extract of aliphatic region **(A)**, and aromatic region **(B)**. **(C)** 2D Score plot of partial least-square discriminant analysis, with components 1 and 2 of multivariate analysis comparing all cell lines. **(D)** VIP scores of the most significantly altered metabolites important for multivariate analysis (*p*-value <0.05).

In order to gather insights into the intracellular metabolism of cells representing different stages of tumor progression, ^1^H NMR spectra of cellular extracts were compared. Tongue normal fibroblasts were also investigated. High quality spectra of at least four replicates from each cell line (ZsG, LN1, and LN2) as well as fibroblasts samples were obtained. All determinations revealed similar spectral characteristics within each cluster of cell line, indicating that metabolic profiles of individual biological replicates were reproducible. Representative 1D ^1^H NMR of the polar phase of the cellular extracts are shown in Figures 1A,B within the range of 0.8 and 8.0 ppm. Assignments of previously identified metabolites were obtained by comparing chemical shifts and spectral peak multiplicities with data from the literature and BMRB (33), HMDB (34), and COLMAR (35) data bases.

The major differences between each step of the metastasis progression, i.e., fibroblasts vs. ZsG, ZsG vs. LN1, and LN1 vs. LN2 were associated to the accumulation or decrease of threonine, threitol, n-acetyl glutamate, methyl malonate, malonate, n-acetyl, creatine, ribose, lactate, ethanolamine, phosphoethanolamine, and unsaturated lipids (Table 1).

**Table 1:** Multivariate analysis data and the highest VIP scores for each comparison (fibroblasts vs. ZsG; ZsG vs. LN1; LN1 vs. LN2).

	Metabolite	δH (ppm)	Status	False discovery rate (FDR) (*q*-value)
Fibroblast vs. ZsG	Threonine	3.59	↓	<1E−15
	Creatine	2.97	↑	1.48E−03
	n-acetyl	2.03	↑	1.86E−03
	Malonate	3.15	↑	1.80E−02
	Ribose + n-acetyl-glutamate	2.21	↑	3.93E−02
	Malonate	3.13	↑	3.93E−02

ZsG vs. LN1	Ribose + n-acetyl-glutamate	2.21	↑	<1E−15
	Threitol	3.69	↑	<1E−15
	Phosphoethanolamine	3.99	↑	8.60E−06
	Ethanolamine	3.83	↑	1.39E−05
	(CH2)n	1.27	↑	1.02E−02
	n-acetyl	2.03	↑	4.35E−02

LN1 vs. LN2	(CH2)n	1.27	↑	4.90E−13
	Ribose + n-acetyl-glutamate	2.21	↓	4.86E−07
	Methyl-malonate	1.21	↓	4.17E−02

*Table shows the metabolites identified, ^1^H chemical shift values in ppm scale (δH) and status represented by arrows ↑↓ indicating increased or decreased metabolites, respectively, based on VIP scores (>1; *p* < 0.05 from Student’s *t*-test and FDR correction *q* < 0.05)*.

Metabolomic profiles comparing the isolated groups of cells showed clearly the discrimination between normal and cancer cells by multivariate analysis PLS-DA score plot and VIP scores (Figures 1C,D). These analyses were validated using cross validation by the leave-one-out method (23–25), revealing an accuracy of −0.58 *R*^2^ (clear variation) of 0.90 and *Q*^2^ (predictive capability) of 0.57 for two components, representing a reliable classification model. In both measures, the value 1 indicates absolute fitting and high predictive power (36).

Several metabolic changes accompany tumorigenesis progression toward metastasis. To evaluate these alterations during the transformation and over tumor development, we previously defined paired comparisons replicating parental and derived cells. Then, we compared the metabolic profiling between each group and according to multivariate analysis PLS-DA, many metabolites were informative to distinguish the clusters. Among these values, we gathered the buckets with the highest VIP scores (>1; *p* < 0.05 from Student’s *t*-test and FDR correction *q* < 0.05) and loading factors, considering the most discriminating power in each the comparison (Table 1; Figure S1 in Supplementary Material).

### Metabolite Accumulation Is due Mainly to Lipid and Nitrogen Metabolism, through Aminoacid Transformation

In order to understand the origin and the biochemical implications of those metabolites, all the enzymes related to their formation were analyzed using the free software REACTOME (26), thus revealing the most representative biochemical pathways involved. The summary of the major findings is shown in Table 2 and Table S1 in Supplementary Material. It is interesting to mention that there is only one metabolic pathway, related to metabolism of amino acids through aldehyde dehydrogenase 6 family member A1 (ALDH6A1), which use NAD^+^ as cofactor, in fibroblasts and ZsG comparison. ZsG vs. LN1 comparison shows that β-oxidation and lipid metabolism are the most important processes in LN1 cells. In the same way, lipid metabolism is highly dependent on NAD^+^, through hydroxyacyl-CoA dehydrogenase/3-ketoacyl-CoA thiolase/enoyl-CoA hydratase [trifunctional protein], alpha subunit (HADHA). A similar pattern is observed in LN1 vs. LN2 comparison. However, the fatty acid synthase (FASN) uses NADPH as cofactor. Also, interactomes of the related enzymes were performed using STRING software (37), showing clusters of acetylase activity and amino acid metabolism for fibroblast vs. ZsG and ZsG vs. LN1 comparisons, connecting lipid metabolism to acetylation in this last comparison. Between LN1 and LN2, there is a single cluster which is related to beta-oxidation (Figure S2 in Supplementary Material). Lipid metabolism appears to be prominently linked to metastatic phenotypes, while cell cycle regulation seems to be a feature of the less invasive cells.

**Table 2:** Enzymes related to metabolite accumulation.

Metabolite	Related enzymes	Cell comparison
Threonine	THNSL1	Fibroblast vs. ZsG
	THNSL2	

Creatine	GAMT	Fibroblast vs. ZsG
	ASL	

n-acetyl	NAT1	Fibroblast vs. ZsG; ZsG vs. LN1
	NAT2	
	NAT8	
	NAT8B	
	GNPNAT1	
	AANAT	
	HGSNAT	
	ESCO1	
	ESCO2	

Malonate	ALDH6A1	Fibroblast vs. ZsG

Ribose + n-acetyl-glutamate	RBKS	Fibroblast vs. ZsG; ZsG vs. LN1; LN1 vs. LN2
	NAGS	

Phosphoethanolamine	ETNK1	ZsG vs. LN1
	ETNK2	

Ethanolamine	PHOSPHO1	ZsG vs. LN1

Threitol	FGGY	ZsG vs. LN1

(CH2)n	Fatty acid synthase	ZsG vs. LN1; LN1 vs. LN2
	HADHA	
	HADHB	

Methyl-malonate	ALDH6A1	LN1 vs. LN2

*Table shows the enzymes related to the accumulation of metabolites and the corresponding cell comparisons*.

### NAD(P)H FLIM Reveals that LN2 Cells Displays Longer Lifetimes

Most of biochemical pathways depend on reactions promoted by the availability of NAD(P)H, including fatty acids metabolism. Thus, the measurements of NAD(P)H free/bound ratios afford clues about enzymatic activities and enhanced processes (38). In this regard, we performed FLIM to evaluate the NADH free to bound ratio in ZsG, LN1 and LN2 cells. To simplify the analysis, we used the phasor plot approach, in which the fluorescence decay in each pixel is plotted on the phasor plot universe (29). With this approach, it is possible to observe that when cells are undergoing glycolysis there is a higher free/bound NAD(P)H ratio. In contrast, when cells have an increment in oxidative phosphorylation (OXPHOS), a lower free/bound NAD(P)H ratio is observed (39).

**Figure 2:**
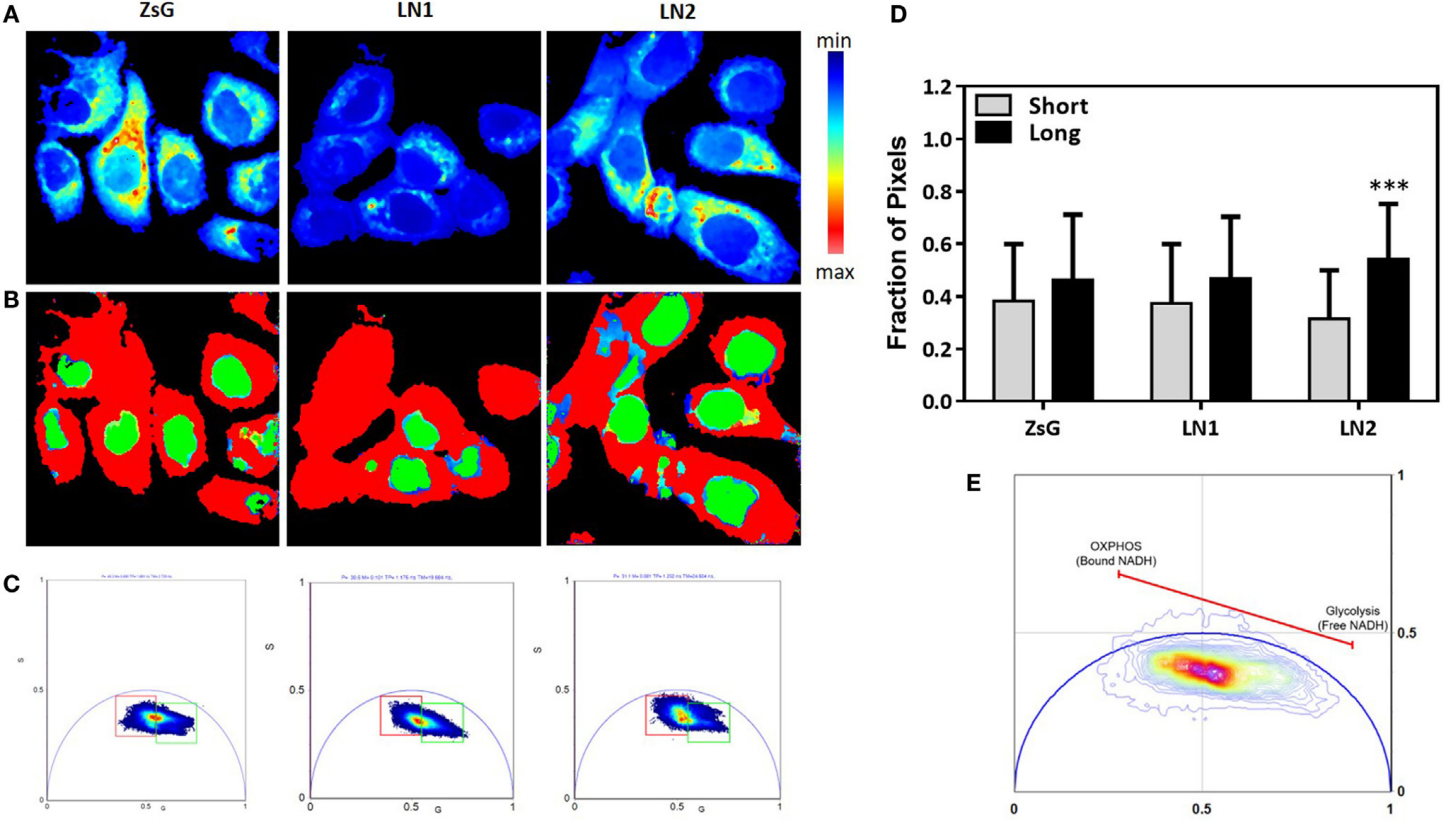
NADH fluorescence lifetime distribution in ZsG, LN1, and LN2 cells. **(A)** Color-coded images generated by the cursor selection according to the phasor plot (colored squares). **(B)** Two-photon fluorescence microscopy intensity images showing NADH fluorescence of ZsG, LN1 and LN2 cells excited at 740 nm. **(C)** Phasor plot illustrating the metabolic trajectories or the fraction of free/bound NADH in FLIM images. **(D)** Fraction of pixels in the phasor plot. **(E)** Metabolic trajectory on the phasor plot. All the images were obtained on a two-photon microscope (Nikon Eclipse TE2000-U) and Plan-Apochromat 60×/1.20 water immersion objective lens (Nikon). Data were acquired on VistaVision software and Alba FLIM instrument (ISS) and analyzed on SimFCS software. Values represent mean ± SD; *N* = 4; ***P < 0.0001; ns compared within each group.

In Figure 2A, the intensity map of NADH fluorescence shows a different distribution of these molecules. To analyze the classical metabolic states (glycolytic and oxidative states), we divided the lifetime distribution of the cells in two cursors, named short and long lifetime cursor (Figures 2B,C). The short lifetime represents a higher NAD(P)H free/bound ratio (colored in red), which means lower binding to dehydrogenases, which is classically associated to a glycolytic profile. The long lifetime represents a lower NAD(P)H free/bound ratio (colored in green). This is related to an increased proportion of binding of NAD(P)H molecules to dehydrogenases, hence associated to OXPHOS (39) (Figure 2E). Moreover, we quantified the percentage of pixels in each image within the cursors and observed that ZsG and LN1 cell lines showed the same pattern of NAD(P)H free/bound ratio whereas LN2 has a lower ratio, indicating that these cell line exhibited a more oxidative profile (Figure 2D).

**Figure 3:**
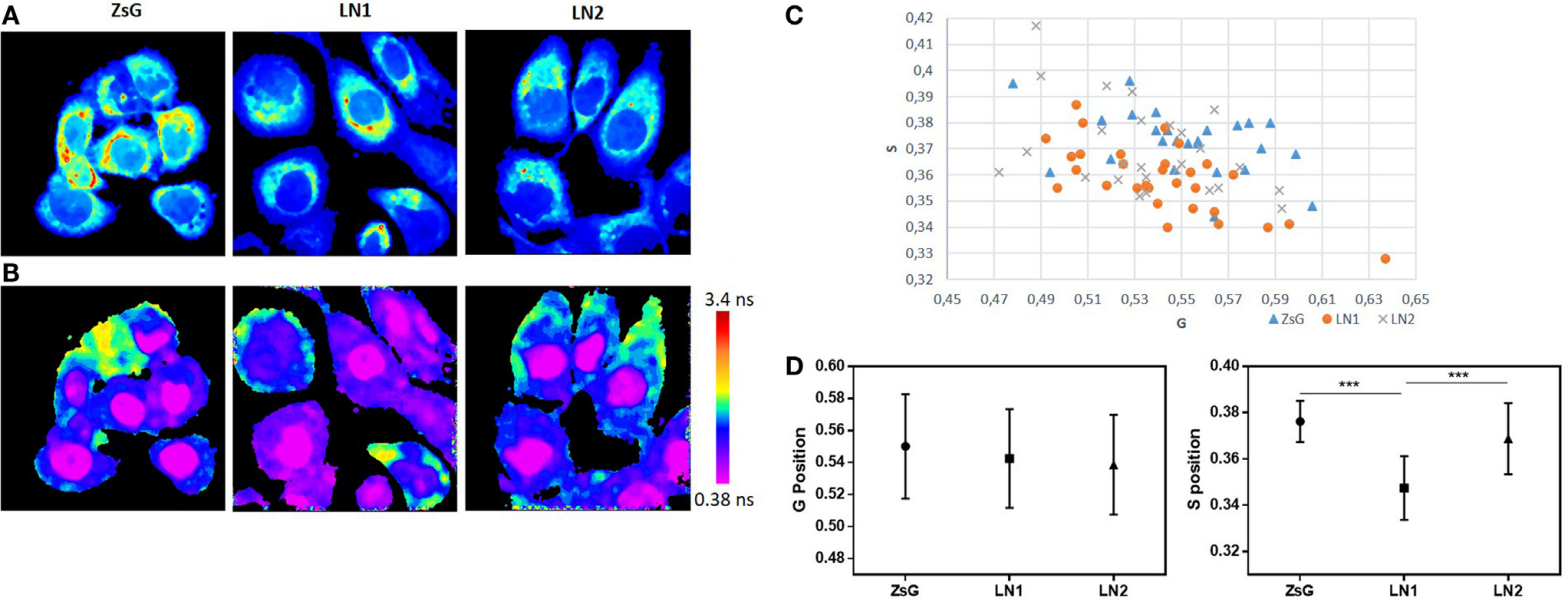
NADH fluorescence lifetime distribution in ZsG, LN1, and LN2 cells. **(A)** Two-photon fluorescence microscopy intensity images showing NADH fluorescence of ZsG, LN1, and LN2 cells excited at 740 nm. **(B)** FLIM map of ZsG, LN1, and LN2 represented by a color-coded scale, by which pixels with a high free/bound NADH ratio are in purple, and pixels with a low free/bound NADH ratio are in red. All the lifetimes ratios are related to a specific color of the scale. **(C)** Phasor fingerprint, representing the mean S and G position of each image phasor distribution. **(D)** Mean S (left) and mean G (right) position of each cell line lifetimes distribution on phasor plot. Values represent mean ± SD; *N* = 5, with five images each; ***P < 0.0001. All the images were obtained on a two-photon microscope (Nikon Eclipse TE2000-U) and Plan-Apochromat 60×/1.20 water immersion objective lens (Nikon). Data were acquired on VistaVision software and Alba FLIM instrument (ISS) and analyzed on SimFCS software.

To better understand and compare the cell lines regarding the lifetime distribution, we analyzed the FLIM maps of each replicate pictured (40). Because the cell lines were shown to be heterogeneous and their phasor plots covered a wide range of different lifetimes, we evaluated the mean phasor plot of each image acquired taking into account all cells present in a certain field in order to obtain the phasor fingerprint of each cell population. Figure 3 shows the distribution regarding means of G and S coordinates of ZsG, LN1, and LN2 cells. Analyzing the metabolic trajectory, we did not observe significant differences between the cell lines regarding the color-coded scale (Figure 3B), the phasor fingerprint (Figure 3C), and also the comparison of the mean G position of each cell line (Figure 3D, left). Notwithstanding, LN1 cells seem to have a dislocated dispersion of lifetimes when compared to ZsG and LN2. This can be seen by analyzing the mean S position of each cell line (Figure 3D, right), in which the pixels of LN1 images have a lower position on the phasor plot. Thus, we observed that although there were no dramatic differences among the cell lines regarding the metabolic pathways (glycolytic vs. oxidative states), they were different when the lifetime distributions were considered.

### Mitochondrial Respiration and Lactate Exportation Does Not Impair the Malignant Transformation in SCC Derived Cell Lines

**Figure 4:**
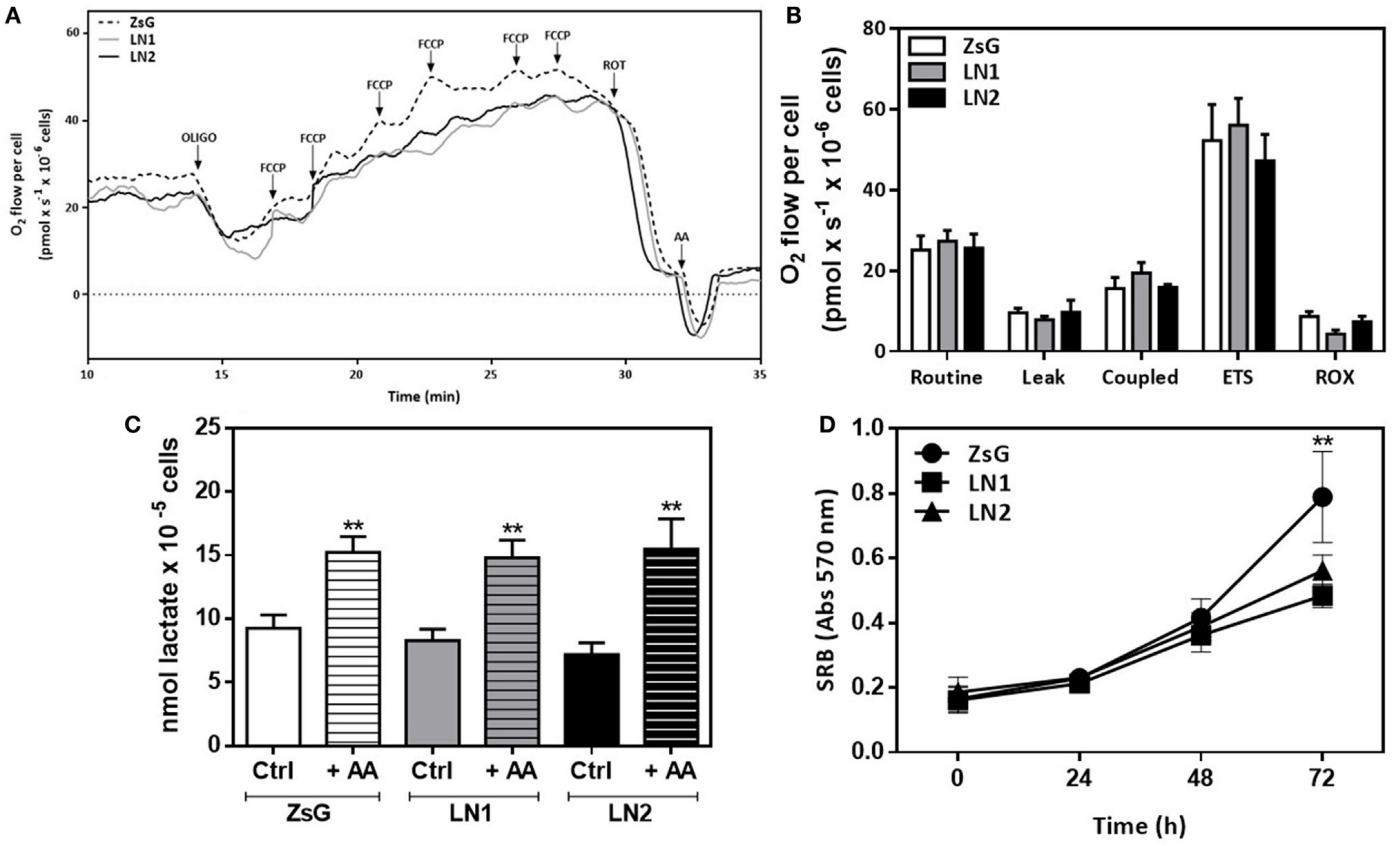
Mitochondrial respiration and lactate exportation does not impair the malignant transformation in squamous cells carcinoma-derived cell lines. High-resolution respirometry assay using Oxygraph-2K (Oroboros Instruments, Innsbruck, Austria) in intact cells. Representative traces of oxygen consumption rate in intact cells **(A)** and absolute results of O_2_ flow per cell (pmol × s^−1^ × 10^−6^ cells) **(B)**. During the assay, cells were maintained in DMEM F12 medium without FBS. Oxygen consumption was evaluated after the addition of modulators of mitochondrial activity [oligomycin, FCCP, rotenone and antimycin A (AA)]. Routine respiration corresponds to endogenous oxygen consumption of cells. All parameters were corrected to ROX values. **(C)** Lactate release after 60 min of incubation with fresh DMEM F12 medium without FBS in the absence (Ctrl) or presence (+AA) of AA. **(D)** Proliferation using sulforhodamine B (SRB) colorimetric assays. Values represent means ± SD; *n* = 4; **P < 0.005 comparing among cell lines (oxygen consumption and proliferation) or within cell line (lactate release).

To further analyze the contributions of oxidative metabolism accompanying the FLIM experiments, we performed high-resolution respirometry assays using OXPHOS modulators in intact cells. Figure 4A displays a representative trace of oxygen consumption rate in each isolated cell line in the presence of culture medium containing the essential substrates (glucose and glutamine). In order to evaluate the dependence of respiratory complexes on oxygen consumption, we used membrane permeable compounds that affect mitochondrial function. Figure 4B shows the absolute values of oxygen consumption showing that it is not possible to distinguish ZsG, LN1, and LN2 by examining their oxidative status.

Metabolomic data showed that cells accumulate lactate during their malignant process (Figure 1D). Moreover, the proliferative phenotype is associated to higher lactate production and transportation to extracellular matrix in keeping with an enhanced glycolytic activity. In order to investigate this feature, we carried out a dynamic lactate liberation assay in presence or absence of AA, which stimulates glycolytic activity. Our results showed that even in the presence of AA there were no differences between all three cell lines (Figure 4C). Additionally, proliferation essays showed that LN1 and LN2 are less proliferative than ZsG cell line (Figure 4D).

## Discussion

The ability of cancer cells to detach from the primary tumor migrate through the blood/lymphatic system and eventually colonize distant tissues must be accompanied by metabolic reprograming. Probing those changes can help to understand the mechanisms of the metastatic processes. Accordingly, we first identified the major metabolites accumulated as the product of metabolic pathways in tongue SCC progression model used here. Taking a holistic approach, we performed ^1^H RMN spectroscopy of ZsG, LN1, and LN2 cell lines as well as normal fibroblasts. We found that the major variations that could be associated to metastasis involved pathways of amino acids and fatty acid metabolism. These were revealed by alterations in the amounts of threonine, ribose, n-acetyl, malonate, methylmalonate, malonate, threitol, n-acetyl glutamate, ethanolamine, phosphoetanolamine, unsaturated lipids (CH_2_)n, and lactate (Figures 1A–D). In order to understand the metabolic pathways related to the principal metabolites accumulated in cells, we carried out paired comparisons considering parental (or one step before in malignant process) and derived cell lines having observed that therein lay the highest differences. Thus, the fibroblasts vs. ZsG, ZsG vs. LN1, and LN1 vs. LN2 cell lines were compared (Table 1; Figure S1 in Supplementary Material). The results in Table 1 show the main differences encountered for metabolites present in the cell lines. The individual occurrence and concentrations of the compounds listed at any given moment could arise from either upstream activation of enzymes participating in the catabolism of various precursors, or from inhibition of downstream enzymes participating in their breakdown. To make a mechanistic sense of these findings, one would have to take into account the fluxes of all involved pathways and then propose models that would fit into coherent biochemical patterns compatible with the phenotypes of the cells displaying different metastatic potentials. Despite the unknown kinetics following the dynamic interplay that contributed toward the pattern observed, the results reflect in reproducible manner instant snapshots of the metabolic status of each cell line. That is, the differences detected are definitely informative in terms of the metabolic reprograming that took place for each cell line. In other words, the results emphasize that the cell lines have individual profiles that can suggest specific ontologies and functional meanings. Nevertheless, it is possible to speculate that some of the compounds listed in Table 1 may actually have roles as intermediaries. This means that in their own right the metabolites could act as effectors participating in regulatory networks, altering the expression patterns and, therefore, impacting cellular plasticity. Among the compounds listed in Table 1, some of the molecules, such as creatine, threitol, threonine, phosphoethanolamine, ethanolamine, and ribose, irrespective of their potential additional roles in other metabolic pathways, could be grouped under the functional class of osmolytes. These are compounds that interchangeably stabilize the intracellular milieu by buffering osmotic perturbations, for instance. The notion that the osmolytes and compatible solutes listed in Table 1 may play such a role opens up the possibility that they could act as effectors of a much broader regulatory network, given their ability to affect flexible and random coil conformations of transcription factors (41–43). In this way, the compounds detected in our metabolomic analyses may have a dual role. On the one hand, they may arise from differentially activated metabolic pathways responding to energy demands and supplying precursors such as building blocks for membranes, DNA, etc. Alternatively, the metabolites could act as bona fide regulators by altering the conformations of members of the signaling pathways and thus generate phenotypes such as the metastatic ones. This model is attractive since one could invoke new levels of regulation that are independent of gain or loss of function induced by those regulations and to grow and survive, cancer cells display alternate metabolic pathways toward tumorigenesis and progression. This hypothesis is currently being investigated in our laboratory.

Further information extracted from the metabolome involved the manual search for enzymes related to metabolite accumulation in their synthesis/degradation using biochemical pathways found in REACTOME free software (Table S1 in Supplementary Material). The results obtained pointed clearly to the involvement of the pathways connected to lipid metabolism. The importance of lipid metabolism becomes apparent when considering that lipid metabolism byproducts can influence growth promoting activity and hence tumorigenesis (44). However, the less invasive cells exhibit enzymes, which although could be broadly included in the lipid metabolism category are associated to specialized reactions pertaining to lipid biochemistry, or perhaps more to the point, catabolism of hydrophobic compounds.

Upon comparison between the metabolomic data between fibroblasts and ZsG, we found that threonine accumulated in fibroblasts. The metabolism of threonine involves the reaction catalyzed by threonine synthase (THNSL1 and THNSL2) forming l-threonine from O-phospho-l-homoserine (45). Threonine biosynthesis initiates from the precursor aspartate leading to a chain of reactions dependent of ATP and NADPH (46). In contrast, ZsG cells accumulated metabolites that promote biosynthesis relying in the reduction of NADP^+^ producing NADPH, such as malonate and ribose. It is worth mentioning that methylmalonate (accumulated in LN1 cells) and malonate are both regulated by ALDH6A1, and that this enzyme is related to fatty acid degradation and amino acid metabolism (Table 2) (47).

Creatine pathway is not related to oxidation/reduction of NAD(P)H despite playing important roles in maintaining storage of ATP in skeletal muscle cells. During muscular contraction, the creatine-phosphate molecules can be decomposed forming ATP before fermentation reestablishes ATP content (48). Creatine may thus be adjuvant in maintaining the demand of ATP (49) in ZsG cell lines. Our data also shows enhanced activity of cell cycle regulation in ZsG and LN1 cells. This has been observed in several cancer cell lines with chromatin cohesion defects, which lead to a poor response to paclitaxel (50). This could mean that ZsG and LN1 cells may be more sensitive to spindle poisons than LN2 cells. Again, the catabolism of some fatty acids such as ω-fatty acids play a major role in the metabolism and biotransformation of exogenous compounds such as xenobiotics. This is achieved for example, by P450 (CYP) mixed function oxidase system that mediates by hydroxylation reactions that in general increase the solubility of these compounds, thus facilitating their excretion. The cytochrome P450 mixed function oxidase enzymes are also known to be involved in the biosynthesis of endogenous substrates such as cholesterol (51).

Phosphoethanolamine and ethanolamine are accumulated in LN1 cells when compared to ZsG. Phospholipids are required to build bilayer membranes in cells, and many studies show that its content increases as long as malignant process arises (52, 53). The membrane components including lipids and proteins allow the formation of ion channels, receptors, and signal transducers important to the interaction of extracellular matrix and cytosol (50), suggesting that those metabolites play important roles in proliferation and growth signaling in LN1 cells.

Comparison between LN1 and LN2 cell lines show that the unsaturated lipids (CH2)n are the only class accumulated in LN2 cells. (CH2)n metabolism shares pathways with valine, leucine, and isoleucine degradation (amino acid metabolism) and fatty acid metabolism. All of them are related to NAD(P)H dynamics. FLIM data confirm these observations (Figure 2D), showing that LN2 cells displays lower NAD(P)H free/bound ratio.

Ribose metabolism is associated to the pentose phosphate pathway (PPP), an important pathway that provides cancer cells with NADPH, ribose-5-phosphate by oxidative pathway. NADPH is essential in the antioxidant defense by glutathione production, while ribose-5-phosphate is an important element for nucleotide biosynthesis. Upregulation of PPP promotes cancer cell survival, proliferation, angiogenesis, invasion and metastasis, and resistance to radiotherapy and chemotherapy. On the other hand, the non-oxidative moiety of PPP reenters fructose-6-phosphate and glyceraldehyde-3-phosphate of the glycolytic pathway, fueling proliferation (54, 55). In the context of our model, the accumulation of ribose was observed in ZsG and LN1 cells. Interestingly, respirometry assays did not show any differences between the cell lines. Plausibly, this could be due to the non-oxidative PPP, fueling the bioenergetic pathways. Furthermore, *N*-acetylglutamate is an obligatory allosteric activator of carbamoyl phosphate synthetase I (CPS-1) (56). CPS-1 is related to cell growth and metabolite levels associated with nucleic acid biosynthesis pathway, as shown by CPS-1 knockdown in lung adenocarcinoma (57). Our analysis revealed that *N*-acetylglutamate is increased in ZsG and LN1 cells, which is compatible with amino acid metabolism and its contributions to nucleotide biosynthesis.

We did not detect differences in lactate exported from cells (Figure 4C) when the three cell lines were compared. The balance between accumulation and exportation lactate in tumor cells has been described as an important factor in regulating glucose metabolism and NAD^+^/NADH availability (58, 59). Much is known about lactate regulation in cancer cells and metabolic switch bearing glycolysis and tumorigenesis (60). Indeed, ZsG cells displaying higher proliferative rates were shown to incorporate less lactate when compared to LN1 and LN2 cells.

The interaction processes between NAD(P)H and proteins was also investigated here. Because these coenzymes play important roles in energy metabolism and, therefore, impact on tumor transformation and progression (20, 38, 61), their participation in the establishment of the metastatic phenotypes was determined, reflecting the importance of measuring NAD(P)H lifetime as an informative biomarker for understanding metabolic reprograming, mitochondrial physiology, oxidative stress, and apoptosis (39). Real-time NAD(P)H lifetime imaging by two-photon fluorescence microscopy allowed prospection into the dynamics of NAD(P)H, by analyzing its binding to specific dehydrogenases, representing an increased NAD(P)H free/bound ratio in glycolytic cells (39). As observed in FLIM experiments, LN2 cells exhibit a relatively smaller free/bound NAD(P)H ratio. However, there were no significant differences when comparing free/bound ratios of ZsG to LN1 cells.

Taken together, the results of the metabolomic analysis and FLIM analysis allowed us to conclude that several pathways connected to lipid metabolism appear to be prominently linked to metastatic phenotypes, while cell cycle regulation and amino acid metabolism are most related to less invasive cells.

## Author Contributions

AS-S and GS contributed to N.M.R. metabolomics performance and analysis; AS-S, AG, and SC contributed to FLIM microscopy performance and interpretation of data; AS-S contributed to respirometry, lactate release, and proliferation assays; AS-S and JP-V contributed to the analyses of metabolic pathways; AS-S, JP-V, GS, SC, and FR discussed the data; FR coordinated the project. All authors participated in the writing and revision process.

## Conflict of Interest Statement

The authors declare that the research was conducted in the absence of any commercial or financial relationships that could be construed as a potential conflict of interest.
