# Supplementary material for: Metabolic Profile of Oral Squamous Carcinoma Cell Lines Relies on a Higher Demand of Lipid Metabolism in Metastatic Cells
Source: Front Oncol. 2018 Feb 2;8:13. doi: 10.3389/fonc.2018.00013 (PMC5801303; doi:10.3389/fonc.2018.00013)
Supplement: Supplementary file 1 [file data_sheet_1.docx]

***Supplementary material***

**Metabolic profile of oral squamous carcinoma cell lines relies on a higher demand of lipid metabolism in metastatic cells.**

**Ana Carolina B. Sant’Anna-Silva^1^, Gilson C. Santos Jr^1,2^, Samir Campos^1^, Juan Alberto Pérez-Valencia^1^, Franklin David Rumjanek^1,*^**

^1^ Instituto de Bioquímica Médica Leopoldo de Meis, Centro de Ciências da Saúde, Universidade Federal do Rio de Janeiro, Rio de Janeiro, RJ, Brazil.

^2^ Centro Nacional de Biologia Estrutural e Bioimagem I (CENABIO I)/Centro Nacional de Ressonância Magnética Nuclear (CNRMN), Laboratório de Ressonância Magnética Nuclear de Biomoléculas (bioNMR), Universidade Federal do Rio de Janeiro, Rio de Janeiro, RJ, Brazil.

**^*^ Correspondence:**

Franklin David Rumjanek

[franklin@bioqmed.ufrj.br](mailto:franklin@bioqmed.ufrj.br)

# Supplementary Figures and Tables

## 1.1 Supplementary Figures


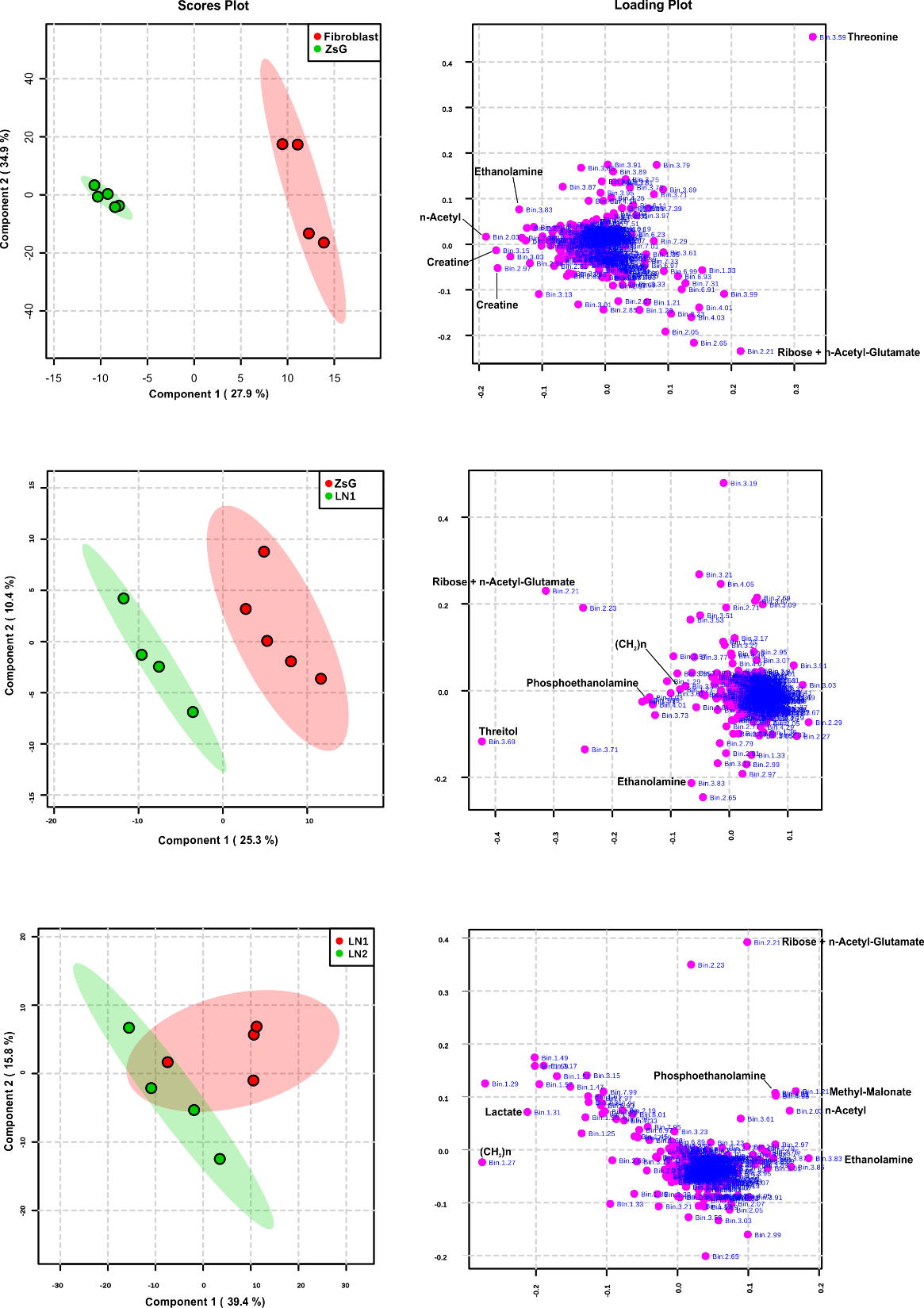


**Supplementary Figure 1:** **Comparison of the supervised (PLS-DA) multivariate analysis in paired groups (fibroblasts *vs*. ZsG ; ZsG *vs*. LN1 ; LN1 *vs*. LN2).** (**A**) PLS-DA score plots in 1 and 2 components showing strong class discrimination and (**B**) PLS-DA loading plot highlighting the important metabolites for class discrimination. Figure was created using the software MetaboAnalyst (Xia *et al.*, 2015).

**
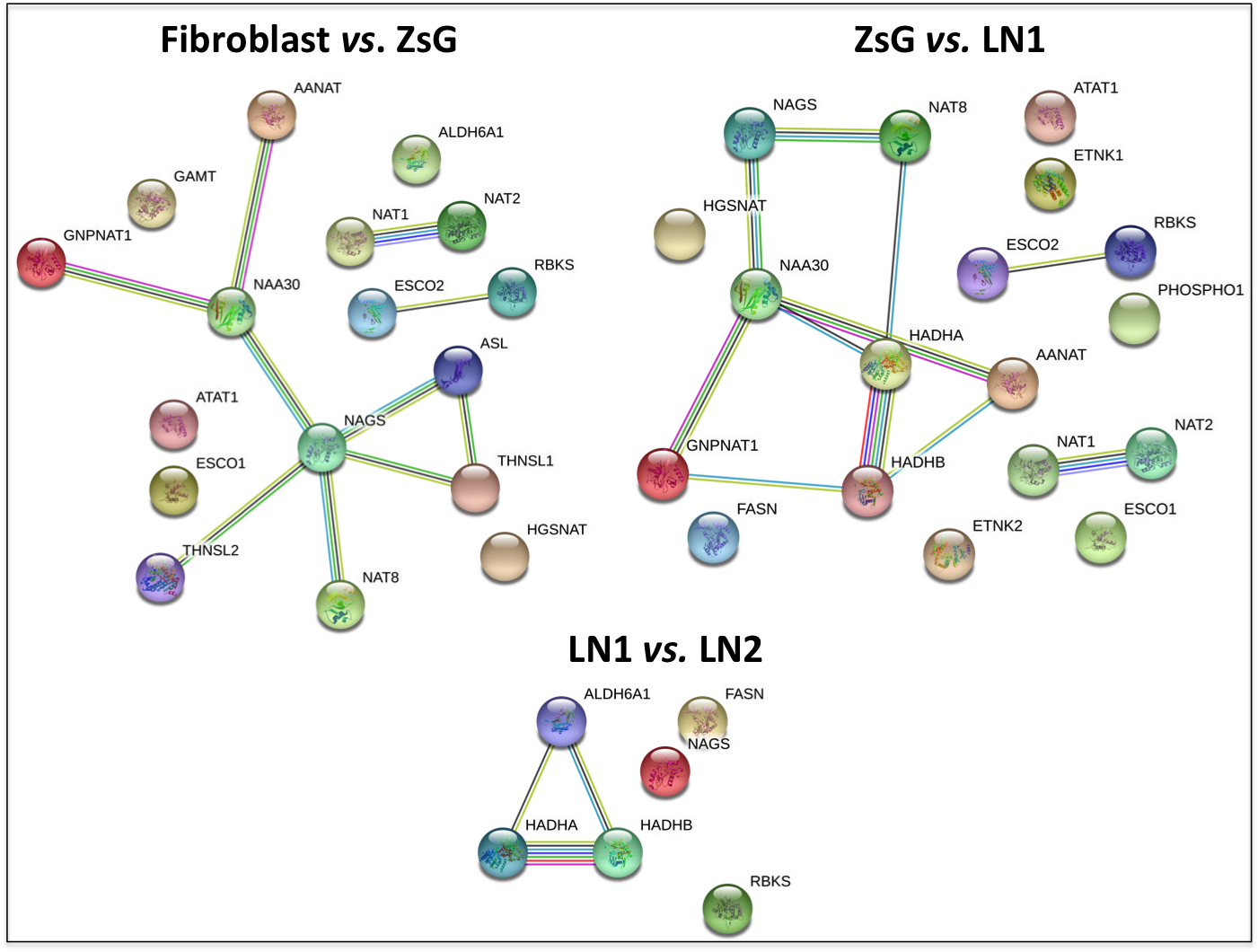
**

**Supplementary Figure 2:** **Interactome showing the contributions of the common enzymes related pathways with metabolites identified.** Figure was created using the free software STRING (Szklarczyk *et al.*, 2017).

**1.1 Supplementary Table**

**Supplementary table 1: Metabolic pathways and related enzymes.** Enzymes associated to biochemical pathways are shown and pathways highlighted depend on NAD(P)^+^/NAD(P)H reactions, emphasizing their cellular activity. FDR correction q<0.05 was used to determine the reliability of related pathways to its metabolites.

|  | **Metabolic pathway** | **FDR (q value)** | **Related enzymes** | **NAD(P)^+^/NAD(P)H cofactor** |
| --- | --- | --- | --- | --- |
| **Fibroblast *vs.* ZsG** | Acetylation | 3,7306E+11 | NAT1 |  |
|  |  |  | NAT2 |  |
|  | Urea cycle | 3,7230E-03 | ASL |  |
|  |  |  | NAGS |  |
|  | Establishment of Sister Chromatid Cohesion | 3,7230E-03 | ESCO1 |  |
|  |  |  | ESCO2 |  |
|  | **Metabolism of amino acids and derivatives** | 5,5087E-03 | GAMT |  |
|  |  |  | ALDH6A1 | NAD^+^ |
|  |  |  | AANAT |  |
|  |  |  | ASL |  |
|  |  |  | NAGS |  |
|  | Metabolism of polyamines | 5,5087E-03 | GAMT |  |
|  |  |  | ASL |  |
|  |  |  | NAGS |  |
|  | Defective SLC6A2 causes orthostatic intolerance (OI) | 1,1547E-02 | NAT1 |  |
|  | MPS IIIC - Sanfilippo syndrome C | 1,1547E-02 | HGSNAT |  |
|  | Amino acid transport across the plasma membrane | 1,1547E-02 | NAT1 |  |
|  |  |  | NAT2 |  |
|  | Astrocytic Glutamate-Glutamine Uptake And Metabolism | 3,2177E-02 | NAT2 |  |
|  | Neurotransmitter uptake and metabolism In glial cells | 3,2177E-02 | NAT2 |  |
|  | Amyloid fiber formation | 3,2177E-02 | NAT8B |  |
|  |  |  | NAT8 |  |
|  | Serotonin and melatonin biosynthesis | 3,2900E-02 | AANAT |  |
|  | SLC-mediated transmembrane transport | 3,2900E-02 | NAT1 |  |
|  |  |  | NAT2 |  |
|  | Synthesis of UDP-N-acetyl-glucosamine | 4,1530E-02 | GNPNAT1 |  |
|  | Transport of inorganic cations/anions and amino acids/oligopeptides | 4,1530E-02 | NAT1 |  |
|  |  |  | NAT2 |  |
|  | Phase II - Conjugation of compounds | 4,1530E-02 | NAT1 |  |
|  |  |  | NAT2 |  |
|  | Creatine metabolism | 4,1530E-02 | GAMT |  |
|  | Mucopolysaccharidoses | 4,1530E-02 | HGSNAT |  |
| **ZsG *vs.*LN1** | Synthesis of phosphatidylethanolamine | 5,7917E-03 | PHOSPHO1 |  |
|  |  |  |  |  |
|  |  |  |  |  |
|  | Glycerophospholipid biosynthesis | 6,1746E-03 | HADHB |  |
|  |  |  |  |  |
|  |  |  |  |  |
|  |  |  |  |  |
|  |  |  |  |  |
|  | Establishment of sister chromatid cohesion | 6,1746E-03 | ESCO1 |  |
|  |  |  | ESCO2 |  |
|  | Acetylation | 6,1746E-03 | NAT1 |  |
|  |  |  | NAT2 |  |
|  | **Beta oxidation of myristoyl-CoA to lauroyl-CoA** | 6,1746E-03 | HADHB |  |
|  |  |  | HADHA | NAD^+^ |
|  | **Beta oxidation of palmitoyl-CoA to myristoyl-CoA** | 6,1746E-03 | HADHB |  |
|  |  |  | HADHA | NAD^+^ |
|  | **Beta oxidation of hexanoyl-CoA to butanoyl-CoA** | 6,1746E-03 | HADHB |  |
|  |  |  | HADHA | NAD^+^ |
|  | **Beta oxidation of lauroyl-CoA to decanoyl-CoA-CoA** | 6,1746E-03 | HADHB |  |
|  |  |  | HADHA | NAD^+^ |
|  | **Beta oxidation of octanoyl-CoA to hexanoyl-CoA** | 6,1746E-03 | HADHB |  |
|  |  |  | HADHA | NAD^+^ |
|  | **Acyl chain remodeling of CL** | 6,1746E-03 | HADHB |  |
|  |  |  | HADHA | NAD^+^ |
|  | **Phospholipid metabolism** | 6,1746E-03 | HADHB |  |
|  |  |  | HADHA | NAD^+^ |
|  |  |  | PHOSPHO1 |  |
|  |  |  | ETNK2 |  |
|  |  |  | ETNK1 |  |
|  | Mitochondrial fatty acid beta-oxidation of unsaturated fatty acids | 6,1746E-03 | HADHB |  |
|  |  |  | HADHA | NAD^+^ |
|  | **Beta oxidation of decanoyl-CoA to octanoyl-CoA-CoA** | 6,1746E-03 | HADHB |  |
|  |  |  | HADHA | NAD^+^ |
|  | Defective SLC6A2 causes orthostatic intolerance (OI) | 3,6502E-02 | NAT1 |  |
|  | **Mitochondrial fatty acid beta-oxidation of saturated fatty acids** | 3,7461E-02 | HADHB |  |
|  |  |  | HADHA | NAD^+^ |
|  | MPS IIIC - Sanfilippo syndrome C | 3,8889E-02 | HGSNAT |  |
|  | Amino acid transport across the plasma membrane | 4,8223E-02 | NAT1 |  |
|  |  |  | NAT2 |  |
| **LN1 *vs.*LN2** | **Activation of gene expression by SREBF (SREBP)** | 1,1077E-03 | FASN | NADPH |
|  | **Regulation of cholesterol biosynthesis by SREBP (SREBF)** | 1,6654E-03 | FASN | NADPH |
|  | **Fatty acid metabolism** | 2,1182E-03 | HADHB |  |
|  |  |  | HADHA | NAD^+^ |
|  |  |  | FASN | NADPH |
|  | **Mitochondrial Fatty Acid Beta-Oxidation** | 2,4709E-03 | HADHB |  |
|  |  |  | HADHA | NAD^+^ |
|  | **Metabolism of lipids** | 4,1368E-03 | HADHB |  |
|  |  |  | HADHA | NAD^+^ |
|  |  |  | FASN | NADPH |
|  | **ChREBP activates metabolic gene expression** | 4,1368E-03 | FASN | NADPH |
|  | **Glycerophospholipid biosynthesis** | 5,3651E-03 | HADHB |  |
|  |  |  | HADHA | NAD^+^ |
|  | **Phospholipid metabolism** | 1,0555E-02 | HADHB |  |
|  |  |  | HADHA | NAD^+^ |
|  | **Metabolism of steroids** | 1,0620E-02 | FASN | NADPH |
|  | Urea cycle | 1,6459E-02 | NAGS |  |
|  | **Vitamin B5 (pantothenate) metabolism** | 1,7990E-02 | FASN | NADPH |
|  | Pentose phosphate pathway (hexose monophosphate shunt) | 2,3587E-02 | RBKS |  |
|  | **Branched-chain amino acid catabolism** | 3,5200E-02 | ALDH6A1 | NAD^+^ |
|  | **Fatty acyl-CoA biosynthesis** | 4,0213E-02 | FASN | NADPH |
|  | **Metabolism of amino acids and derivatives** | 4,0990E-02 | ALDH6A1 | NAD^+^ |
|  |  |  | NAGS |  |

**REFERENCES**

Szklarczyk, D. *et al.* (2017) ‘The STRING database in 2017: quality-controlled protein-protein association networks, made broadly accessible’, *Nucleic acids research*, 45(D1), pp. D362–D368. doi: 10.1093/nar/gkw937.

Xia, J. *et al.* (2015) ‘MetaboAnalyst 3.0-making metabolomics more meaningful’, *Nucleic Acids Research*, 43(W1), pp. W251–W257. doi: 10.1093/nar/gkv380.
